# Cellular Assays for High-Throughput Screening for Modulators of Trk Receptor Tyrosine Kinases

**DOI:** 10.2174/1875397300801010027

**Published:** 2008-02-25

**Authors:** Jun Wang, Michael K Hancock, Jeanne M Dudek, Kun Bi

**Affiliations:** Invitrogen Corporation, Discovery Sciences, 501 Charmany Drive, Madison, WI 53719, USA

## Abstract

Trk receptor tyrosine kinases are required for signal transduction initiated by neurotrophins leading to cell proliferation, differentiation, survival and death. Alterations in Trk kinase activity have been linked to various diseases. To address the need for cell-based assays for screening and studying the selectivity of Trk kinase modulators, we developed high-throughput cell-based assays for Trk receptor kinases using nuclear factor of activated T-cells (NFAT) beta-lactamase reporter lines stably expressing full length human Trk kinases. These assays were functionally validated with cognate neurotrophin(s), inhibitors and TRK RNAi oligos and demonstrated for their utility in identifying potent and selective modulators of Trk receptor kinases.

## INTRODUCTION

Neurotrophin signaling plays an essential role in neuronal and non-neuronal cell proliferation, differentiation, survival and death. Alterations in neurotrophin signaling have been linked to neurodegenerative disorders such as Alzheimer’s disease, psychiatric disorders such as depression, as well as various cancers including neuroblastoma [1, 2]. Neurotrophins are a family of growth factors including nerve growth factor (NGF), brain-derived neurotrophic factor (BNDF), neurotrophin 3 (NT-3) and neurotrophin 4 (NT-4) [1]. Each neurotrophin can bind and activate two types of cell surface receptors – the Trk family receptor tyrosine kinases (TrkA, TrkB and TrkC) and the p75 neurotrophin receptor (p75^NTR^). p75^NTR^ binds all neurotrophins with a similar affinity, whereas the Trk receptors are selective for different neurotrophins. NGF binds preferentially to TrkA; BDNF and NT-4 to TrkB; NT-3 preferably to TrkC receptor. Unlike other neurotrophins, NT-3 is promiscuous and can also bind TrkA and TrkB with lower affinity at least in certain cell types [3]. The binding of neurotrophins to their cognate Trk receptor leads to Trk dimerization and autophosphorylation. The phosphorylated receptors then recruit and increase the phosphorylation of phospholipase C-γ (PLC-γ), Src and Shc, which leads to the activation of PI3K, ERK and PKC/Ca^2+^ pathways. These events in turn result in the activation of transcription factors such as cyclic AMP-response element binding protein (CREB) and nuclear factor of activated T-cells (NFAT) leading to downstream gene expression [4]. The importance of Trks in neurotrophin signaling has made Trk kinases candidate targets for several therapeutic areas. Constitutively active oncogenic forms of TrkA and TrkC have been found in patients with neuroblastoma and leukemia [5, 6]. There are currently few specific Trk inhibitors available for therapy. Similar to CEP701, K252a, an indole carbazol compound of microbial origin, has been widely used as a Trk inhibitor [7]. It also inhibits many other kinases including mixed-lineage kinase 3 (MLK3) [8].

The lack of specific Trk inhibitors is partially due to the lack of robust cell-based assay tools for high-throughput screening. The most commonly used assays for Trk activity include low-throughput receptor autophosphorylation analysis by western blotting [9] and luciferase-based reporter assays for TrkA and B [10]. Here, we described the development of cell based assays for all three Trk kinases applicable to high-throughput screening. Human full length TrkA, B and C cDNAs were each stably integrated into a NFAT beta-lactamase (NFAT-*bla*) reporter line. Single clones for each Trk were isolated by fluorescence-activated cell sorting (FACS) based on their response to neurotrophins. Each cell line was validated with the cognate ligand(s), known inhibitors and TRK specific RNAi oligos. The selectivity and robustness of our reporter assays for Trk kinases indicate that these assays are suitable for high-throughput screening of Trk modulators as well as determining the selectivity of compounds against three Trk kinases.

## PRINCIPLE OF ASSAY DESIGN

**Fig. (1):**
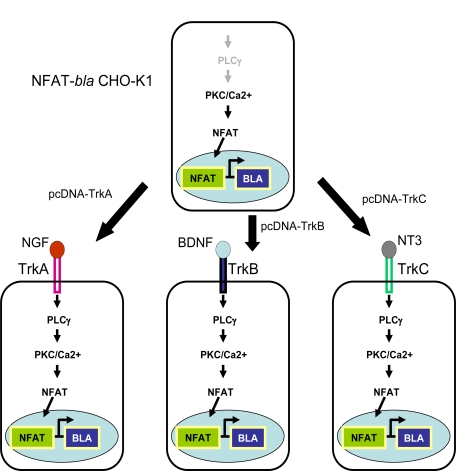
Diagram of the cell line design for Trk receptor kinases.

Expression vectors carrying full length human TrkA, TrkB or TrkC were each stably transfected into NFAT-*bla* CHO-K1 reporter line characterized previously (Fig. **1**). Single clone for each Trk was isolated after FACS sorting. Ligand binding to its cognate receptor (NGF for TrkA, BDNF for TrkB and NT-3 for TrkC) leads to the activation of PLC-γ/PKC/Ca^2+^ pathway, which in turn activates transcription factor NFAT. Activated NFAT translocates to the nucleus and bind to its DNA response elements driving the expression of downstream reporter gene, in this case, beta-lactamase (Fig. **1**). Beta-lactamase activity can be detected by adding its substrate, CCF4-AM, directly onto cells [11]. In the absence of neurotrophins, the pathway is not active therefore there is little beta-lactamase activity. When cells are loaded with CCF4-AM and are excited at 406 nm, the substrate emits at around 530 nm. When cells are stimulated with neurotrophin, the pathway is activated, leading to the beta-lactamase reporter activation. The loaded substrate is then cleaved by beta-lactamase, disrupting the fluorescence resonance energy transfer (FRET) and resulting in emission at 460 nm with excitation at 406 nm.

## METHODS

### Generation of Stable Cell Clones

Full length human TRKA, TRKB and TRKC cDNAs were synthesized by Blue Heron Biotechnology (Bothell, MA) according to the sequences of NM_002529 for TRKA, NM_006180 for TRKB and NM_002530 for TRKC, and cloned into pcDNA6.2/V5-DEST (Invitrogen, Carlsbad, CA) via Gateway™ LR reaction.

NFAT beta-lactamase reporter line NFAT-*bla* CHO-K1 cells (Invitrogen, Carlsbad, CA, catalog number K1078) were transfected using Lipofectamine™ LTX transfection reagent (Invitrogen, Carlsbad, CA) with pcDNA6-TRKA, pcDNA6-TRKB and pcDNA6-TRKC and selected for blasticidin-resistance. Blasticidin-resistant TrkA transfected cells were stimulated with NGF (Invitrogen, catalog number 13257-019), TrkB transfected cells with BDNF (Invitrogen, catalog number PHC7074) and TrkC transfected cells with NT-3 (Invitrogen, catalog number PHC7034) for 5 hours before neurotrophin stimulated beta-lactamase expressing cells were collected by FACS as single cells onto 96-well plates. The survived clones that responded to the respective ligand were selected and expanded. The clone with the best response to each ligand was further tested in a 384-well format for the dose response of neurotrophins and inhibitors and in a 96-well format for RNAi knock-down.

### Neurotrophin Stimulation and 384-Well Beta-Lactamase Assay Protocol

Cells in a sub-confluency state (or cryopreserved cells) were resuspended in Assay Medium (DMEM with GlutaMAX™ (Invitrogen, catalog number 10569) supplemented with 0.1% dialyzed FBS, 0.1 mM NEAA, 25 mM HEPES, 100 U/mL Penicillin and 100 µg/mL Streptomycin) and plated in a 384-well assay plate (Corning, Lowell, MA, catalog number 3712) at 10,000 cells (32 μL) per well. 32 μL of Assay Medium without cells were plated in Cell-free Control wells on the same plate. 4 µL 1% DMSO was added to each well to reach final DMSO concentration of 0.1% to mimic the screening situation where compounds dissolved in DMSO are added to test wells. Cells were then stimulated with 4 µL/well 10 x ligands (NGF, BDNF, NT-4 (Invitrogen, catalog number PHC7024), NT-3 or Thapsigargin (Sigma, St. Louis, MO)) over the indicated concentration range (1 pM ~ 50000 pM) for 5 hours before adding 8 µL/well 6X LiveBLAzer™-FRET B/G Substrate mixture (Invitrogen, catalog number K1096) for 2 hours. Fluorescence intensity at excitation 406 nm and emission 460 nm and 530 nm were obtained using Tecan Safire^2^ fluorescence plate reader (Tecan, Durham, NC) (Table **1**). After subtracting the average fluorescence intensity from the Cell-free Control wells, the 460nm/530nm emission ratio was calculated. Response Ratio is a measurement of the assay window and is calculated as the 460nm/530nm Emission Ratio of the stimulated wells divided by the 460nm/530nm Emission Ratio of the unstimulated wells. Response Ratios were plotted against test ligand concentrations in log scale and then analyzed using Prism software (GraphPad Software, Inc. San Diego, CA). Sigmoidal dose-response equation with varying slope was used to fit the data and generate EC_50_ values. Z’-factor values were calculated as: Z’-factor = 1 - [(3 x stdev_unstim_ + 3 x stdev_maxstim_) / (avg_maxstim_ – avg_unstim_)].

### Inhibitor Treatment

Cells were seeded into a 384-well assay plate at 10,000 cells/well as described above and treated for 30 minutes with 4 µL of AG879 (EMD, San Diego, CA, catalog number 658460), or GW441756 (Tocris Bioscience, Ellisville, MO, catalog number 2238) or K252a (Invitrogen, catalog number PHZ1131), each at 10 x final concentrations. Cells were then stimulated with 4 µL of neurotrophins at 10 x EC_80 _concentrations (EC_80_ concentrations are: NGF, 0.77 nM; BDNF, 1 nM; NT-3, 0.72 nM; Thapsigargin, 8 nM) for 5 hours before beta-lactamase assay was performed as described above.

### RNAi Experiment

Cells were plated in 96-well assay plates (Corning, catalog number 3603) at 6,000 cells/well in 100 µL growth medium (DMEM containing 10% dialyzed FBS, 0.1 mM NEAA and 25 mM HEPES), and incubated in 37°C, 5% CO_2_ incubator overnight. Next morning, Stealth™ RNAi (Invitrogen, catalog number 10620318) and Lipofectamine^TM ^RNAiMAX (Invitrogen, catalog number 13778) complexes were prepared according to the manufacturer’s protocol and added to the wells (50 nM RNAi oligos and 1µL RNAiMAX/well). RNAi oligo targeting for beta-lactamase was used as the positive control and a random-sequenced oligo with 48% GC content (Med GC) as the negative control. A set of 2 oligos for each Trk was used:

TRKA1 (ACAUCAUCGAGAACCCACAAUACUU)

TRKA2 (ACGCUGCUCCUUGUGCUCAACAAAU)

TRKB1 (UGGUAAUGCUGUUUCUGCUUAAGUU)

TRKB2 (ACACCACGAACAGAAGUAAUGAAAU)

TRKC1 (GCCAAGUGUAGUUUCUGGCGGAUUU)

TRKC2 (CCAGACCAAUCUGAACUGGACCAAU)

Cells were incubated with RNAi oligos at 37°C for 32 hours, followed by a medium change. Cells were incubated in Assay Medium at 37ºC for 16 hours and then stimulated with respective ligands (NGF, 0.77 nM; BDNF, 1 nM; NT-3, 0.72 nM and Thapsigargin, 30 nM) for 5 hours before beta-lactamase assay was performed as described above.

## RESULTS AND DISCUSSION

### Assay Development and Validation

**Fig. (2):**
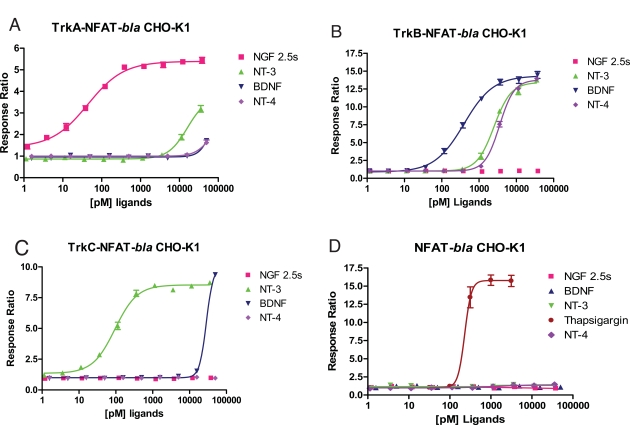
**Neurotrophin dose responses of TrkA, TrkB, TrkC and parental NFAT-*bla* CHO-K1 cell lines.** TrkA-NFAT-*bla* CHO-K1 (**A**), TrkB-NFAT-*bla* CHO-K1 (**B**), TrkC-NFAT-*bla* CHO-K1 (**C**), and parental NFAT-*bla* CHO-K1 (**D**) cells were stimulated with NGF (■), BDNF (▲), NT-4 (♦), NT-3 (▼) and Thapsigargin (●, parental cells only) over the indicated concentration range in the presence of 0.1% DMSO for 5 hours before beta-lactamase assay was performed as described in *Methods*. Response Ratios were plotted for the indicated concentrations of each ligand (n=5 for each data point).

For the purpose of developing cell-based assays for each Trk kinase, we generated stable clones of NFAT-*bla* CHO-K1 expressing full length human TrkA, TrkB or TrkC as described in *Methods*. Each clone was validated for the dose response to NGF, BDNF, NT-3 and NT-4 (Fig. **2**, Table **2**). TrkA-NFAT-*bla* CHO-K1 cells responded well to NGF with a maximum response ratio of 5.5 fold (Z’ factor of 0.75) and an EC_50_ of 0.044 nM, consistent with previously published results [12]. TrkA-NFAT-*bla* CHO-K1 cells showed a 3-fold response over baseline to NT-3 with an EC_50_ around 16 nM, suggesting that NT-3 binds TrkA with a lower affinity than NGF [13]. BDNF and NT-4 had a minimal effect on TrkA-NFAT-*bla* CHO-K1 cells, but significantly stimulated beta-lactamase reporter activity in TrkB-NFAT-*bla* CHO-K1 cells with a maximum response ratio of 14 (Z’ value of 0.82) and an EC_50_ of 0.39 nM for BDNF and a maximum response ratio of 13 (Z’ value of 0.8) and an EC_50_ of 3.5 nM for NT-4 (Fig. **2B**). TrkB-NFAT-*bla* CHO-K1 cells also responded to NT-3 with a maximum response ratio of 13 and an EC_50_ of 2.5 nM, but did not respond at all to NGF, which is in agreement with previous findings [13]. TrkC-NFAT-*bla* CHO-K1 cells responded to TrkC preferred ligand NT-3 with a maximum response ratio of 8.5 (Z’ value of 0.9) and an EC_50_ of 0.093 nM (Fig. **2C**). TrkC-NFAT-*bla* CHO-K1 cells responded to BDNF only at the highest concentration (50 nM) tested and did not show any response to NGF and NT-4. All three cell lines responded to thapsigargin treatment with a similar EC_50_ (data not shown) to that of the parental NFAT-*bla* CHO-K1 cells (Fig. **2D**). Taken together, TrkA-NFAT-*bla* CHO-K1 cells specifically responded to TrkA preferred neurotrophin, NGF, TrkB-NFAT-*bla* CHO-K1 cells to BDNF, NT-3 and NT-4, and TrkC-NFAT-*bla* CHO-K1 cells to TrkC ligand NT-3. The fact that NT-3 generated dose responses in both TrkC and TrkB cells with a 10 fold less EC_50_ in TrkC cells confirms that NT-3 binds to both TrkC and B with TrkC being the preferred receptor. The fact that CHO cells lack p75^NTR^ expression [14] and the parental NFAT-*bla* CHO-K1 cells do not respond to neurotrophins (Fig. **2D**) suggests that the effect we observed for each neurotrophin was mediated by the engineered Trk receptor. Our results confirmed differential affinity and rank order potency of each neurotrophin to Trk receptors as reported in the literature, which suggests that these cell-based reporter assays for Trk kinases can be used for studying the potency and selectivity of Trk modulators.

### Inhibitor Activities

**Fig. (3):**
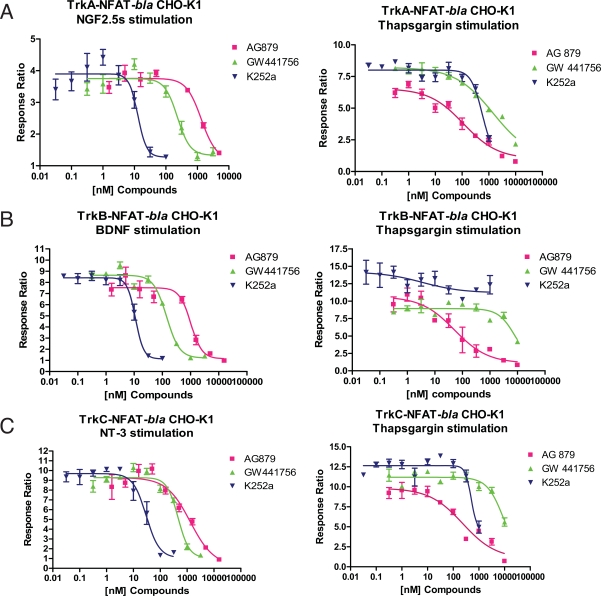
Inhibition of neurotrophin-induced beta-lactamase reporter activity in the cognate Trk receptor expressing cells. Cells were pretreated with indicated concentrations of K252a (▼), or AG879 (■) and GW441756 (▲) and then stimulated with NGF (TrkA cells, **A**), BDNF (TrkB cells, **B**), or NT-3 (TrkC cells, **C**), or Thapsgargin for all three cell lines for 5 hours before beta-lactamase assay was performed as described in *Methods*. Response Ratios were plotted for the indicated concentrations of each compounds (n=3 for each data point).

We further validated these assays with a commonly used Trk kinase inhibitor, K252a. Dose response experiment showed K252a inhibited NGF-induced beta-lactamase activity in TrkA-NFAT-*bla* CHO-K1 cells (Fig. **3A**), BDNF-induced beta-lactamase activity in TrkB-NFAT-*bla* CHO-K1 cells (Fig. **3B**) and NT-3 induced TrkC-NFAT-*bla* CHO-K1 cells (Fig. **3C**) with low nano-molar IC_50_ values (Table **3**), which are in accordance with literature values using various types of assays [7, 15, 16]. Interestingly, K252a had a partial inhibitory effect on Thapsgargin induced reporter activity in all three cell lines at the highest concentration tested (Fig. **3**, Table **3**). AG879, also known as an EGFR family kinase inhibitor [17], has been reported in the literature to inhibit NGF induced TrkA autophosphorylation and downstream PLC-γ phosphorylation in micro-molar concentrations [18]. When tested in our reporter assays, AG879 inhibited all three Trk-mediated NFAT activation with an IC_50_ around 1 μM (Table **3**). Interestingly, this compounds inhibited Thapsigargin-induced NFAT reporter activity with much lower IC_50_s, suggesting that AG879 affects other targets than Trks. GW441756, a potent TrkA inhibitor in a biochemical assay [19], also inhibited all three Trk kinases induced NFAT-*bla* reporter activity with an IC_50 _value of 0.23 μM for TrkA, 0.14 μM for TrkB and 0.46 μM for TrkC. It also affected Thapsigargin-induced NFAT reporter activity, but only at much higher concentrations (Fig. **3**, Table **3**). These data suggest that GW441756 is a more potent and selective Trk inhibitor than AG879. Our data provides first evidence that GW441756 is a potent inhibitor for all three Trk receptor kinases. This inhibitor experiment demonstrated how one can use these three cell assays to identify compounds based on both potency and selectivity.

### RNAi Validation

**Fig. (4):**
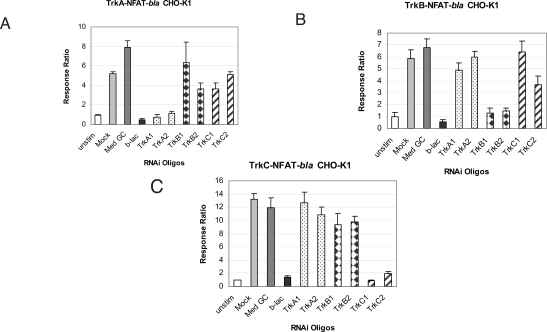
Effects of various RNAi oligos on neurotrophin-induced beta-lactamase reporter activity. Cells were incubated with indicated RNAi oligos for 32 hrs, followed by a medium change and additional 16 hour-incubation. Cells were then stimulated with respective ligands (NGF for TrkA cells, **A**; BDNF for TrkB cells, **B**; NT-3 for TrkC cells, **C**) for 5 hours before beta-lactamase assay was performed as described in *Methods*. Response Ratios were plotted for each RNAi oligo (n=3 for each data point). Unstim: untransfected cells were left unstimulated with ligands; Mock: cells were treated with transfection reagent (no RNAi oligo) and stimulated with ligands.

To further demonstrate the specificity of each assay, we examined the effect of Trk specific RNAi oligos on neurotrophin-induced NFAT-*bla* reporter activity. We also determined the specificity of each oligo by examining the effect of the oligos on thapsigargin-induced reporter activity. Cells were treated with Stealth™ Select RNAi oligos and stimulated with neurotrophins as described in *Methods*. Beta-lactamase gene-specific RNAi (b-lac) was used as a positive control and showed complete inhibition of neurotrophin-induced reporter activity in all three cell lines (Fig. **4**). RNAi oligo with 48% GC content (Med GC) was used as a negative control and had no inhibitory effect in any of the three assays. Treatment with two TrkA RNAi oligos significantly knocked down NGF induced beta-lactamase activity in TrkA expressing cells (Fig. **4A**) and had little effect on BDNF induced beta-lactamase activity in TrkB cells (Fig. **4B**) and NT-3 induced in TrkC cells (Fig. **4C**). Treatment with the two TrkB oligos completely inhibited BDNF induced reporter activation in TrkB cells (Fig. **4B**) and had minimal effect on the TrkA and TrkC cells induced by NGF and NT-3 respectively (Fig. **4A** and **C**). Finally, treatment with the two TrkC oligos completely knocked down NT-3 induced beta-lactamase reporter activity in TrkC cells and had minimal inhibitory effect on TrkA and TrkB cells (Fig. **4C**). Longer incubation (72 hour) with these RNAi oligos gave a similar inhibitory pattern (data not shown). No cytotoxicity effect was observed with any of the RNAi oligos used even after 72 hour incubation.

**Fig. (5):**
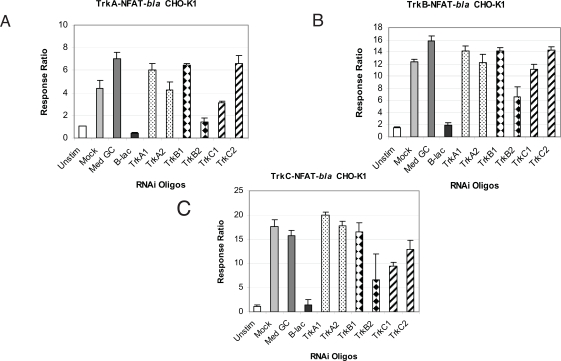
Effects of various RNAi oligos on thapsigargin-induced beta-lactamase reporter activity. TrkA-NFAT-*bla* CHO-K1 (**A**), TrkB-NFAT-*bla* CHO-K1 (**B**) and TrkC-NFAT-*bla* CHO-K1 (**C**) cells were incubated with indicated RNAi oligos for 32 hrs, followed by a medium change and additional 16 hour-incubation. Cells were then stimulated with thapsigargin for 5 hours before beta-lactamase assay was performed as described in *Methods*. Response Ratios were plotted for each RNAi oligo (n=3 for each data point). Unstim: untransfected cells were left unstimulated with ligands; Mock: cells were treated with transfection reagent (no RNAi oligo) and stimulated with thapsigargin.

To further address the specificity of these RNAi oligos, we examined the effect of these oligos on thapsigargin-induced NFAT-*bla* reporter activity. Thapsigargin works downstream of the Trk receptors by directly increasing intracellular Ca^2+^ concentration leading to the activation of NFAT independent of Trk activity. Accordingly, specific Trk RNAi oligos should not knockdown thapsigargin induced reporter activity. Indeed, the two TrkA, TRKC2 and TRKB1 RNAi oligos did not inhibit thapsigargin effect in all three cell lines (Fig. **5**). TRKB2 oligo and TRKC1 to a less extent, however, had about 50% inhibitory effect in all three cell lines stimulated with thapsigargin, suggesting that TRKB2 and TRKC1 may have effects on other targets that contribute to thapsigargin induced-signaling.

## CONCLUSIONS

In conclusion, we have developed NFAT reporter assays that can be used for high-throughput screening for modulators of Trk receptor tyrosine kinases. Taking the advantages of the beta-lactamase reporter which allows for a ratiometric and sensitive read-out, these assays are an improvement over previously described luciferase-based assays [10], which were shown robust in a 96-well format. Our assays are easy to set up, can be miniaturized to a 384-well format and take less than one working day to run. Most importantly, our assays are selective and robust with Z’ factors great than 0.7, pico-molar detection sensitivity and large assay windows (5 to 14 folds). The selectivity and specificity of these assays were demonstrated by the EC_50_ results of the four neurotrophins and receptor RNAi knockdown experiments. The K252a, AG879 and GW441576 inhibitor study demonstrated the utility of these cell assays for determining compound potency and selectivity and for compound library screening against TrkA, TrkB and TrkC.

## Figures and Tables

**Table 1:** Beta-Lactamase Assay Protocol for Trk-NFAT-*bla* CHO-K1 Cells in 384-Well Format

Step	Parameter	Value	Description
1	Cells	32 µL	Trk-NFAT-*bla* CHO-K1 cells, 10,000 cells/well
2	DMSO or Compound	4 µL	10 X compound solution or 1% DMSO
3	Neurotrophin	4 µL	10 X Neurotrophin
4	Incubation	5 hrs	37^o^C, 5% CO_2_
5	Substrate	8 µL	6 X CCF4–AM
6	Incubation	2 hrs	Room Temp.
7	Detection	fluorescence	Tecan Safire^2^

**Table 2:** EC_50_ Values of Neurotrophins in the Three Trk Assays

Neurotrophins (nM)	TrkA-NFAT-*bla* CHO-K1	TrkB-NFAT-*bla* CHO-K1	TrkC-NFAT-*bla* CHO-K1
NGF 2.5s	0.04	>50	>50
BDNF	>50	0.39	28*
NT-4	>50	3.5	>50
NT-3	16*	2.5	0.09

* Values may not be accurate as the curves did not reach saturation.

**Table 3:** IC_50_ Values (in nM) of Inhibitors in the Three Trk Assays

	TrkA		TrkB		TrkC	
	NGF	Thaps	BDNF	Thaps	NT-3	Thaps
K252a	13	570*	11	>1000	30	500*
AG879	1326	112	1011	51	1308	231
GW44176	231	2831*	141	8205*	460	8970*

* Values may not be accurate as the curves did not reach saturation.

## References

[1] Chao MV, Rajagopal R, Lee FS (2006). Neurotrophin signaling in health and disease. Clin Sci.

[2] Chao M (2003). Neurotrophins and their receptors: A convergence point for many signaling pathways. Nat Rev Neurosci.

[3] Barbacid M (1994). The Trk family of neurotrophin receptors. J Neurobiol.

[4] Groth RD, Coicous LG, Mermelstain PG, Seybold VS (2007). Neurotrophin activation of NFAT-dependent transcription contributes to the regulation of pro-nociceptive genes. J Neurochem.

[5] Schramm A, Schulte JH, Astrahantseff K, Apostolov O, Limpt V, Sieverts H, Kuhfittig-Kulle S, Pfeiffer P, Versteeg R, Eggert A (2005). Biological effects of TrkA and TrkB receptor signaling in neuroblastoma. Cancer Lett.

[6] Liu Q, Schwaller J, Kutok J, Cain D, Aster JC, Williams IR, Gilliland DG (2000). Signal transduction and transforming properties of the TEL-TRKC fusions associated with t(12;15)(p13;q25) in congenital fibrosarcoma and acute myelogenous leukemia. EMBO J.

[7] Berg MM, Sternber DW, Parada LF, Chao MV (1992). K-252a inhibits nerve growth factor-induced trk-proto-oncogene tyrosine phosphorylation and kinase activity. J Biol Chem.

[8] Roux PP, Dorval G, Boudreau M, Angers-Loustau A, Morris SJ, Makkerh J, Barker PA (2002). K252a and CEP1347 are neuroprotective compounds that inhibit mixed-lineage kinase-3 and induce activation of Akt and ERK. J Biol Chem.

[9] Miller SG (2000). Discovery of cytokine mimics using cell-based systems. Drug Discov Today.

[10] Zhang J, Chen D, Gong X, Ling H, Zhang G, Wood A, Heinrich J, Cho S (2006). Cyclic-AMP response element-based signaling assays for characterization of Trk family tyrosine kinases modulators. Neurosignals.

[11] Zlokarnik G, Negulescu PA, Knapp TE, Mere L, Burres N, Feng LX, Whitney M, Roemer K, Tsien RY (1998). Quantitation of Transcription and Clonal Selection of Single Living Cells with Beta-Lactamase as Reporter. Science.

[12] Ahamed J, Venkatesha RT, Thangam EB, Ali H (2004). C3a enhances nerve growth factor-induced NFAT activation and chemokine production in a human mast cell, line HMC-1. J Immunology.

[13] Ryden M, Ibanez CF (1996). Binding of neurotrophin-3 to p75NTR, TrkA, and TrkB mediated by a single functional epitope distinct from that recognized by TrkC. J Biol Chem.

[14] Zapf-coby Z, Olefsky J (1998). Nerve growth factor processing and trafficking events following trkA-mediated endocytosis. Endocrinology.

[15] Groth RD, Mermelstein PG (2003). Brain-derived neurotrophic factor activation of NFAT (nuclear factor of activated T-cells)-dependent transcription: A role for the transcription factor NFATc4 in neurotrophin-mediated gene expression. J Neurosci.

[16] Rende M, Brizi E, Conner J, Treves S, Censier K, Provenzano C, Taglialatela G, Sanna PP, Donato R (2000). Nerve growth factor (NGF) influences differentiation and proliferation of myogenic cells *in vitro via* TrkA. Int J Dev Neurosci.

[17] Zhou Y, Li S, Hu YP, Wang J, Hauser J, Conway AN, Vinci MA, Humphrey L, Zborowska E, Wilson JKV, Brattain MG (2006). Blockade of EGFR and ErbB2 by the novel dual EGFR and ErbB2 kinase inhibitor GW572016 sensitizes human colon carcinoma GEO cells to apoptosis. Cancer Res.

[18] Ohmichi M, Pang L, Ribon V, Gazit A, Levitzki A, Saltiel AR (1993). The tyrosine kinase inhibitor tyrophostin blocks the cellular actions of nerve growth factor. Biochemistry.

[19] Wood ER, Kuyper L, Petrov KG, Hunter RN, Harris PA, Lackey K (2004). Discovery and *in vitro* evaluation of potent TrkA kinase inhibitors: oxindole and aza-oxindoles. Bioorg Med Chem Lett.

